# Vaping for weight control: Findings from a qualitative study

**DOI:** 10.1016/j.abrep.2020.100275

**Published:** 2020-04-28

**Authors:** Fiona Dobbie, Isabelle Uny, Sarah E. Jackson, Jamie Brown, Paul Aveyard, Linda Bauld

**Affiliations:** a Usher Institute and SPECTRUM Consortium, College of Medicine and Veterinary Medicine, University of Edinburgh, UK; b Institute for Social Marketing, University of Stirling, UK; c Department of Behavioural Science and Health and SPECTRUM Consortium, University College London, UK; d Nuffield Department of Primary Care Health Sciences, University of Oxford, UK

**Keywords:** E-cigarettes, Vaping, Weight control, Weight loss, Qualitative research

## Abstract

**Introduction:**

Smokers have expressed concern about weight gain once they stop smoking and weight gain is a risk factor associated with smoking relapse. Nicotine in e-cigarettes, as well as vaping behaviour, may support smoking cessation by reducing weight gain. This study explored the factors that influence attitudes towards, and awareness of, e-cigarettes and weight control post smoking cessation.

**Methods:**

Qualitative study involving focus groups with adults in the UK (n = 58) who were either exclusive vapers or dual users.

**Results:**

There was limited awareness and/or inclination to vape to prevent weight gain after stopping smoking. Reasons for this centred on: the health gains of stopping smoking outweighing any potential weight gain; a lack of understanding of the appetite supressing effects of nicotine; a belief that vaping could not suppress appetite like a cigarette and could result in craving for certain flavours; concerns about the longer-term effects of e-cigarettes on health and the ethics of promoting vaping as way to support smoking cessation by limiting weight gain, especially for young women.

**Conclusion:**

Participants in this study do not appear inclined to use e-cigarettes to prevent weight gain after smoking cessation. There is a lack of understanding about why nicotine might help prevent weight gain and a concern that e-cigarette flavours could provoke cravings and that vaping may be unsafe in the long-term.

## Highlights

• Awareness or inclination to vape for weight control was low among participants.
• Vapers may unconsciously use their device instead of snacking.
• Understanding nicotine consumption and its association with weight control was low.
• Further evidence is needed to assess vaping for weight control.

## 1 Introduction

Smokers tend to weigh less than non-smokers. This difference is mostly likely due to the appetite suppressing effects of nicotine and the hand-to-mouth action of smoking that may influence food consumption and prevent or delay weight gain (Cena et al., 2011, Freathy et al., 2011, Winsløw et al., 2015). People who smoke have expressed a belief that smoking helps them control their weight, but this varies by gender. For example, a large survey of adolescents in the USA found that girls were twice as likely as boys to report smoking to lose or control their weight, particularly when they considered themselves to be overweight (Fulkerson & French, 2003).

It is therefore not surprising that smokers express concern about gaining weight once they stop smoking (Landrau-Cribbs, Cabriales, & Cooper, 2015). A recent systematic review and *meta*-analysis found that the average quitter gains up to 4.8 kg within the first year and that a significant percentage of quitters (13%) gain in excess of 10 kg (Aubin et al., 2012, Lycett et al., 2011). It has also been reported that the decrease in smoking prevalence observed in some countries may have contributed to the rise in obesity (Courtemanche, Pinkston, Ruhm, & Wehby, 2016). The potential for weight gain is a barrier to smoking cessation (Aubin et al., 2012, Lycett et al., 2011), particularly for smokers who are already more likely to be concerned about their weight (e.g. girls with overweight, people with an eating disorder and pregnant women) (Beebe and Bush, 2015, Morean and L'Insalata, 2018). Futher, some evidence shows that larger weight gain is associated with an increased risk of smoking relapse (Borrelli & Mermelstein, 1998) while other studies report contradictory findings (Hall, Tunstall, Vila, & Duffy, 1992).

In some countries, e-cigarettes have become popular aids to smoking cessation. For example, e-cigarettes were used in more than one nearly a third of quit attempts in England in 2014 (West, Shahab, & Brown, 2016). There is growing interest in whether the nicotine in e-cigarettes, as well as the ‘*ritualistic behaviours’* of vaping (e.g. refilling an e-cigarette and continuing the hand-to-mouth action typically associated with smoking) could support smoking cessation and prevent relapse by reducing associated weight gain (Glover, Breier, & Bauld, 2016). Recent studies have found that women are more likely than men to use e-cigarettes to manage their weight and moods (Piñeiro et al., 2016, Strong et al., 2015) and that female smokers with overweight or obesity are more likely than men to experiment with e-cigarettes (Cox et al., 2019, Strong et al., 2015). One study found that some e-cigarette users report using sweet flavoured e-liquids on occasion to replace sweet foods (Cox et al., 2019). There is also evidence to suggest that smokers who switch to vaping may limit post-cessation weight gain at least in the short term (Russo et al., 2016) and that this phenomenon requires further exploration.

Members of our team (Jackson et al., 2019) previously conducted a cross-sectional survey of English smokers and e-cigarette users to vaping for weight control. This nationally representative, interviewer administered, survey of past-year and current smokers and e-cigarette users in England (n = 1439, conducted in 2018) explored relevant attitudes, beliefs and behaviours. It concluded that not only was the prevalence of e-cigarette use to control weight low, so too was awareness of any potential link between vaping and weight control.

This paper presents findings from a qualitative study that was conducted in parallel with the survey and seeks to ‘unpack’ survey results and add to currently limited evidence exploring the potential of vaping for weight control. It will address two key questions: 1) Why was awareness and use of vaping for weight control so low? 2) What factors influenced attitudes towards future vaping to control weight?

## 2 Method

### 2.1 Research design and sample

This was a qualitative (focus group) study, conducted in 2018, with UK adults who were either exclusive vapers or dual users. Topic guides were informed by findings from the survey that preceded the qualitative study (Jackson et al., 2019). They were developed by two researchers (IU, FD) and then reviewed by the authors and finalised. A purposive quota sampling frame was developed to provide diversity of age, gender, ethnicity, and employment status. Participants were recruited from central Scotland in two ways. First, two researchers (IU, FD) attended an event organised by the New Nicotine Alliance (a UK not for profit vaping advocacy organisation) and compiled a list of people who were interested in taking part. Second, a dedicated Facebook page and a Facebook advert were created to promote and recruit for the study for three months to the general population in the study area (July to Sept 2018). The Facebook page reached 1384 people, 197 of whom engaged directly on the page via comments and direct messages about their experiences of weight loss, vaping and smoking cessation. The advert reached 20,546 people in total over the three months. To recruit people aged 18–29 years, we used Twitter combined with the Facebook page and posters at one UK University (Stirling).

These recruitment methods resulted in a sample of 58 participants. 55 of these individuals took part in focus groups organised into eight single-sex groups. Three participants could not attend scheduled focus group meetings and were interviewed separately (one individual interview and one paired interview). Care was taken not to over recruit from the NNA (we did not want to bias the sample with experienced vapers who may have been very knowledgeable about the products and how to vape). The majority of participants were recruited through Facebook and Twitter (n = 50) with eight from NNA. All participants provided fully informed consent. Ethical approval was granted by the University of Stirling General University Ethics Panel (GUEP324). Findings are reported using the Standards for Reporting Qualitative Research (SRQR) guidelines (O’brien, Harris, Beckman, Reed, & Cook, 2014).

### 2.2 Analysis

Focus groups were recorded and transcribed verbatim. Immediately after each focus group, an observation sheet was completed to record initial observations and points of interest for context and analysis. Transcripts were analysed using a thematic approach, facilitated by NVivo 11. A coding frame was developed by reading transcripts to identify the key topics and issues which emerged from data. Next, a draft analytical framework was created, piloted, refined and finalised by three authors (IU, LB, FD). Each transcript was then coded and summarised into key themes by IU and FD. This approach allowed for emergent patterns and explanations to be explored and created a platform for movement beyond what Braun and Clarke (2006) refer to as the *‘surface of the data’* (i.e. descriptive analysis) and into interpretive analysis.

### 2.3 Findings

Findings focused on three main themes: 1) attitudes to post-cessation weight gain; 2) awareness and experience of e-cigarettes for weight control; 3) attitudes towards vaping for future weight control

Table 1 presents the participant characteristics by focus group. Participants in all groups included a mix of ex-smokers who were now exclusively vaping and dual users (continuing smokers who also vape).

**Table 1:** Participant characteristics by focus group.

Group number	Age	Number of participants	
		Female	Male
1	18–29	9	0
2	18–29	0	9
3	30–44	8	0
4	30–44	0	7
5	45–54	4	0
6	45–54	0	6
7	55+	8	0
8	55+	0	7

### 2.4 Attitudes to post-cessation weight gain

The starting point for analysis was to understand whether participants were concerned about gaining weight when they stopped smoking cigarettes. Focus group findings suggested this was not a major concern, with participants tending to accept that some weight gain was to be expected, but that this was outweighed by the longer-term health benefits of stopping smoking.

“*I wasn’t that concerned about it because I would rather not smoke. I would rather be overweight than have a heart attack or whatever*.” (Group 1 Female aged 18–29)

There was also a view that weight gain was driven by other factors, regardless of smoking behaviour.

*“ I never, ever, ever had to worry about my weight until I was into my 40 s, and I slowly started putting on a wee belly. But I wouldn’t put that down to stopping smoking. It’s just turning 40, 41, 42, 43.”* (Group 6 Male aged 45–54)

For those who did express concern around post-cessation weight gain this differed by gender. Women, particularly younger women, were more likely to be concerned than men, and men were more likely to believe that women would be more concerned about weight gain than them.

*(Interviewer) Do any of you have any concerns about weight gain when you stop smoking?*

*Female 1: Yeah.*

*Female 2: See I’ve just stopped, we stopped at the same time and all I do is eat now.*

*Female 3: Same. I’ve already put on like half a stone.*

Although a number of men, usually older men, had noticed that they had put on more weight after quitting smoking, on the whole, men appeared to be less concerned and were of the view that excess weight could controlled through exercise, diet and will power.

*“I’ve started going to the gym and that… When I was smoking, I would wake up in the morning and feel a tight chest and stuff. Then that kinda eased once I stopped. And then, I went to a gym and that. People had said to me when you stop smoking you can put on weight and that – I’m like, you just need to control it.*” (Group 6 Male aged 45–54)

### 2.5 Awareness and experience of e-cigarettes for weight control

Findings suggested that participants had limited awareness and/or inclination to vape to prevent weight gain after stopping smoking. Participants tended to cite other reasons for switching to vaping: it was perceived to be more socially acceptable than smoking cigarettes; offered the ability to control the amount of nicotine intake; was viewed as a hobby; provided enjoyment through the community and social side of vaping; and was seen as a healthier and cheaper alternative to cigarettes.

However, there were examples of participants who purposefully chose to vape as a means to keep their hands busy or curb their snacking:

*“I definitely think it’s a good option because it keeps the mouth habit going so instead of grabbing like a packet of crisps, and constantly eating them, or chocolates, or anything like that, it’s the sweetness as well. It kind of subsides any hunger and stuff like that.*” (Group 2 Male aged 18–29)

The above quote also highlights the importance of flavours and sweetness of e-liquid to vapers, which supports a view that vaping could potentially offer similar levels of satisfaction to food but without the additional energy. (Cox et al., 2019).

### 2.6 Factors that influence attitudes and use of e-cigarettes

Awareness and use of e-cigarettes to prevent post-cessation weight gain may be explained by several factors identified during the study. These have been summarised under three themes: understanding the biology of nicotine; unintentional vaping for weight control; and uncertainty that vaping could prevent weight gain.

#### 2.6.1 Understanding the biology of nicotine

Focus groups participants reported a limited awareness that nicotine was an appetite suppressant. It was discussed in all groups but only three groups raised it spontaneously. This may contextualise the lack of concern about weight gain - if participants did not know that the nicotine in their cigarette could suppress their appetite, why would they be concerned about putting on weight if they stopped smoking? Thus, it is understandable that, given this knowledge gap, there was a lack of interest in vaping to maintain nicotine levels and potentially prevent weight gain.

#### 2.6.2 Unintentional vaping for weight control

A lack of conscious awareness of vaping for weight control does not mean it was completely absent. Participants suggested it may have been unconscious.

*“It kind of unconsciously helps. You never sit there and you’re like I don’t want to eat so I’m going to vape but you do it without really thinking about it sometimes I reckon.*” (Group 2 Male aged 18–29)

One participant explained how he accidentally discovered that vaping could help him control his weight. This participant identified as an ex-smoker who used to smoke occasionally until he started to vape and then gave up cigarettes completely. He discovered vaping through his wife who was bored with available e-liquid flavours so he decided to make his own for her to try. In doing so, he started to try the flavours and realised that not only did he enjoy vaping, but that it also helped him control his weight

*“This time last year I was nearly 22 stone. I was the heaviest I’ve ever been in my life. I’m now just under 18. And I have kept that weight off through vaping* … *I’ve got that many flavours in the house that I’ve made up* … *So I’ll pick that up and vape that. So it gets rid of that sensation of I’ll need to go and have a cup of tea and a biscuit. I’ll still have a cup of tea. But I don’t need to have anything to eat with it.*” (Group 6 Male 45–54)

What is interesting about this account is that this participant did not intentionally vape to lose weight. Rather, by creating new flavours for his partner he (inadvertently) discovered that vaping could help to control his own weight.

#### 2.6.3 Uncertainty that vaping could prevent weight gain

Participants were sceptical that vaping could aid post-cessation weight control. This was discussed in three ways. First was the view that vaping different flavours could result in a craving for the particular food being vaped.

*“But then that can go the opposite way because if you’re smoking something that tastes of a particular flavour you then get that craving for that particular type of food.*” (Group 1 Female 18–29)

Participants did not believe that vaping could suppress their appetite to the same extent as smoking because they were aware that stopping smoking reinstated a sense of taste, increasing enjoyment of food, creating the potential to eat more.

*“I have put on weight but that’s…I’m just being awful greedy now. I cannae help myself. I phone up and order all sorts of food now just because you’ve got your taste buds back so…*” (Group 4 Male 30–44)

The final perspective was that vaping to control post-cessation weight could not be a long-term solution and was therefore pointless, with participants expressing the view that people would be better off dealing with their weight gain sooner rather than later

*“It just isn’t a long-term solution like if you want to keep the weight off, vaping is not the answer* … *Because if you’re going to eventually stop vaping you may as well just stop smoking and deal with the weight gain then instead of in 5 years or whatever.*” (Group 1 Female 18–29)

### 2.7 Attitudes towards vaping for future weight control

Given the lack of awareness and understanding of the appetite supressing effects of nicotine, it is not surprising that participants tended to view vaping for weight control with caution. This was discussed in three ways. First was the perceived risk of vaping for a prolonged period, especially when the evidence regarding longer-term use is in its infancy.

*“I’m still quite concerned about the long-term impact on my health because, well as far as I was aware there wasn’t that much research, because it’s still quite a new thing.*” (Group 3 Female 30–44)

The second reason centred on the ethical concerns of promoting vaping as way to support smoking cessation by limiting weight gain. While there was support for promoting vaping to stop people smoking cigarettes, making the leap to vaping as a tool to prevent weight gain was too much for some participants who favoured more established methods such as exercise and diet.

*“I would say recommend e-cigarettes to get off smoking, fine. But for the subsequent weight loss surely you would recommend and promote things, exercise, diet, change in lifestyle.*” (Group 2 Male 18–29)

A strong view was that any public health message about vaping for weight control would need to be very clear to protect vulnerable adolescents who may start to vape purely to control their weight (i.e. not to support a quit attempt). A number of younger women in the 18–29 focus group expressed this concern. This was especially poignant when one participant shared that she and her friends had originally taken up smoking to curb their eating and thus control their weight.

*“All 4, myself included, of the girls that I lived with, either still do or have had eating disorders and their… all of their reasons for taking up smoking other than the social aspects was to replace eating.*” (Group 1 Female 18–29)

The final view was a concern that vaping could be just as addictive (or more so) as cigarettes due to the fact that vapers could vape continuously, which is in contrast to a cigarette, which has a start and finish.

*“I’m just a little bit worried about almost how frequent[ly] I use it [e-cigarette] because of how addicted I am. Like right now like I wish I could just keep using it.*” (Group 2 Male 18–29)

## 3 Discussion

This exploratory study aimed to identify whether a phenomenon highlighted in the literature (vaping for weight control) was indeed taking place in practice in the UK. Findings from this qualitative study, in combination with a recent published cross-sectional survey (Jackson et al., 2019), indicate that awareness and use of e-cigarettes for post-cessation weight control is not common, as has been highlighted in other recent studies (Rhoades et al., 2019). While there were some examples from focus group participants who had substituted vaping for eating or snacking, this was not the key motivation for vaping and often resulted more from an unconscious or coincidental action.

Awareness of vaping for weight control may be low for at least two reasons. First the rise of e-cigarettes, especially in the UK, has occurred at a time when a large proportion of adults are already overweight or obese. This may undermine any link between vaping and weight control. Second, while it is plausible that vaping could aid weight control (e.g. via effects of nicotine on appetite and behavioural substitution) there is currently little evidence that vaping helps to prevent post-cessation weight gain. Findings from this study highlighted that this lack of evidence was an important factor in shaping public opinion towards vaping for weight control. Focus group participants had doubts that vaping could act as an appetite suppressant and were uncertain as to how it could be used as tool to prevent the weight gain associated with smoking cessation. There was a particular concern about the potentially addictive nature of vaping, and also the risks for vulnerable groups (e.g. those with an eating disorder). The potential and risks of vaping for weight control in vulnerable populations have been reported in a number of recent studies (Landrau-Cribbs et al., 2015, Miller et al., 2017, Napolitano et al., 2018, Strong et al., 2015), particularly around young people, those suffering from anxiety and other psychological disorders, and those with (actual or perceived) overweight or obesity.

Findings from this study add to our understanding of smokers’ awareness of and attitudes towards vaping for weight control. First, despite concerns about post-cessation weight gain being present (especially for women), this was viewed as inevitable and outweighed by the health benefits of giving up cigarettes. This suggests that smokers who want to stop smoking would not take up vaping purely to prevent any potential weight gain. Furthermore, there was a lack of understanding of the appetite suppressing effects of nicotine, and nicotine in general as previous studies have found (King et al., 2018, Wilson et al., 2019). Participants held a belief that cigarettes may prevent weight gain but they did not necessarily know that this was due to the nicotine in cigarettes. Should any future smoking cessation initiative seek to encourage smokers to switch to an e-cigarette to help them control their weight, it would need to clearly explain the link between nicotine and weight in order for smokers to understand the rationale.

Future studies, particularly randomised controlled trials, could assess and report weight as a secondary outcome to yield further evidence about the link, if any, between vaping and weight control post-cessation. This could be particularly relevant for studies with longer-term follow up (Russo et al., 2016). Further research is also merited to explore different participant characteristics with respect to attitudes, knowledge and awareness of issues to do with weight and vaping. This would include gender and age but also other socio-demographic characteristics, as e-cigarettes expectancies, e-cigarette use and motivations for using an e-cigarette for smoking cessation may vary between groups. In addition, further research should explore how the variety of e-liquid flavours may play a role, given preliminary evidence from other studies that vapers may replace consuming sweet or sugary snacks with flavours that mimic confectionary or other sugar-sweetened food products (Cox et al., 2019).

A strength of this study is its use of qualitative methods which adds greater depth of understanding to an under researched area. However, a limitation of qualitative methods is that findings are not generalizable to the wider vaping/dual use community. Nonetheless, the qualitative findings from this study are strengthened by similar findings from the quantitative study in which the qualitative component was situated.

## 4 Conclusion

Our findings provide little support for the promotion of e-cigarettes to prevent weight gain following smoking cessation. Further evidence is required to assess whether e-cigarettes can prevent weight gain associated with smoking cessation. If further research generates new evidence to show that vaping should be promoted to prevent weight gain post smoking cessation, awareness raising measures will be required to explain the effects of nicotine on appetite and weight and any behavioural aspects linked to vaping that may affect weight. ​The associated ethical considerations would require careful thought, especially for adolescents and non-smokers.

## Role of Funding Sources

This work was supported by a 10.13039/501100000289TAG Cancer Research UK grant (grant number C22086/A24778). Wider data collection for the Smoking Toolkit Study and SEJ and JB’s salaries were also funded by Cancer Research UK (grant number C1417/A22962). LB and JB are co-investigators in the SPECTRUM Consortium that receives funding from the UK Prevention Research Partnership, an initiative funded by UK Research and Innovation Councils, the Department of Health and Social Care (England) and the UK devolved administrations, and leading health research charities. PA is an NIHR senior investigator and funded by NIHR Oxford Biomedical Research Centre and CLAHRC. The funders played no role in study design, collection, analysis, or interpretation of data, writing the manuscript, and the decision to submit the manuscript for publication.

## 6 Contributors

JB, PA, FD, SJ and LB designed the study and acquired funding. FD and IU conducted analyses. FD, IU and LB wrote the first draft of the manuscript. All authors contributed to and have approved the final manuscript.

## Declaration of Competing Interest

JB has received unrestricted research funding relating to smoking cessation from Pfizer, who manufacture smoking cessation medications. All authors declare no financial links with tobacco companies or e-cigarette manufacturers or their representatives.
